# Supplementary material for: The Effect of Acute Oral Galactose Administration on the Redox System of the Rat Small Intestine
Source: Antioxidants (Basel). 2021 Dec 24;11(1):37. doi: 10.3390/antiox11010037 (PMC8773155; doi:10.3390/antiox11010037)
Supplement: Supplementary file 1 [file antioxidants-11-00037-s001.zip › antioxidants-1495961-supplementary.pdf]

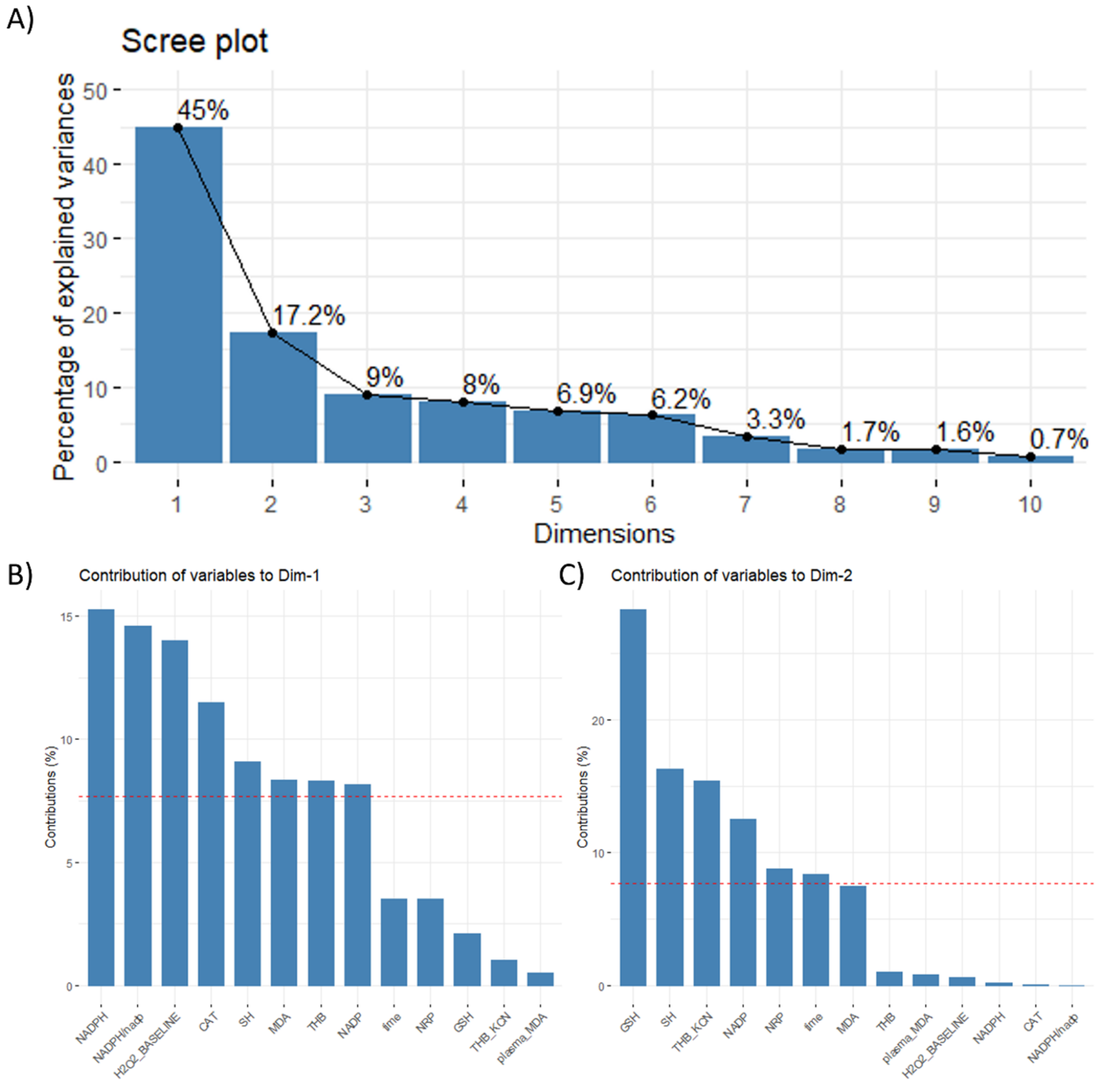

Figure S1. Combined intestinal redox-related parameters principal component analysis. (**A**) Scree plot. (**B**) Contribution of individual variables to the 1st principal component. (**C**) Contribution of individual variables to the 2nd principal component. NRP – nitrocellulose redox permanganoemtry; time – minutes since the exposure to treatment (continuous variable); THB_KCN – change in 1,2,3-trihydroxybenzene autooxidation rate in the presence of 2mM potassium cyanide (KCN); GSH – glutathione (the main representative of the low molecular weight thiols (LMWT)); MDA – malondialdehyde (the main representative of the thiobarbituric acid reactive substances (TBARS)); NADP – nicotinamide adenine dinucleotide phosphate; THB – change in 1,2,3-trihydroxybenzene autooxidation rate; CAT – catalase activity (hydrogen peroxide dissociation rate); H2O2_BASELINE – estimated baseline levels of tissue hydrogen peroxide; SH – protein reactive sulfhydryl groups; NADPH – reduced nicotinamide adenine dinucleotide phosphate; NADPH/nadp – the concentration of reduced nicotinamide adenine dinucleotide phosphate corrected for concentration of nicotinamide adenine dinucleotide phosphate; contrib – contribution of variables; Dim1 – 1st dimension; Dim2 – 2nd dimension.

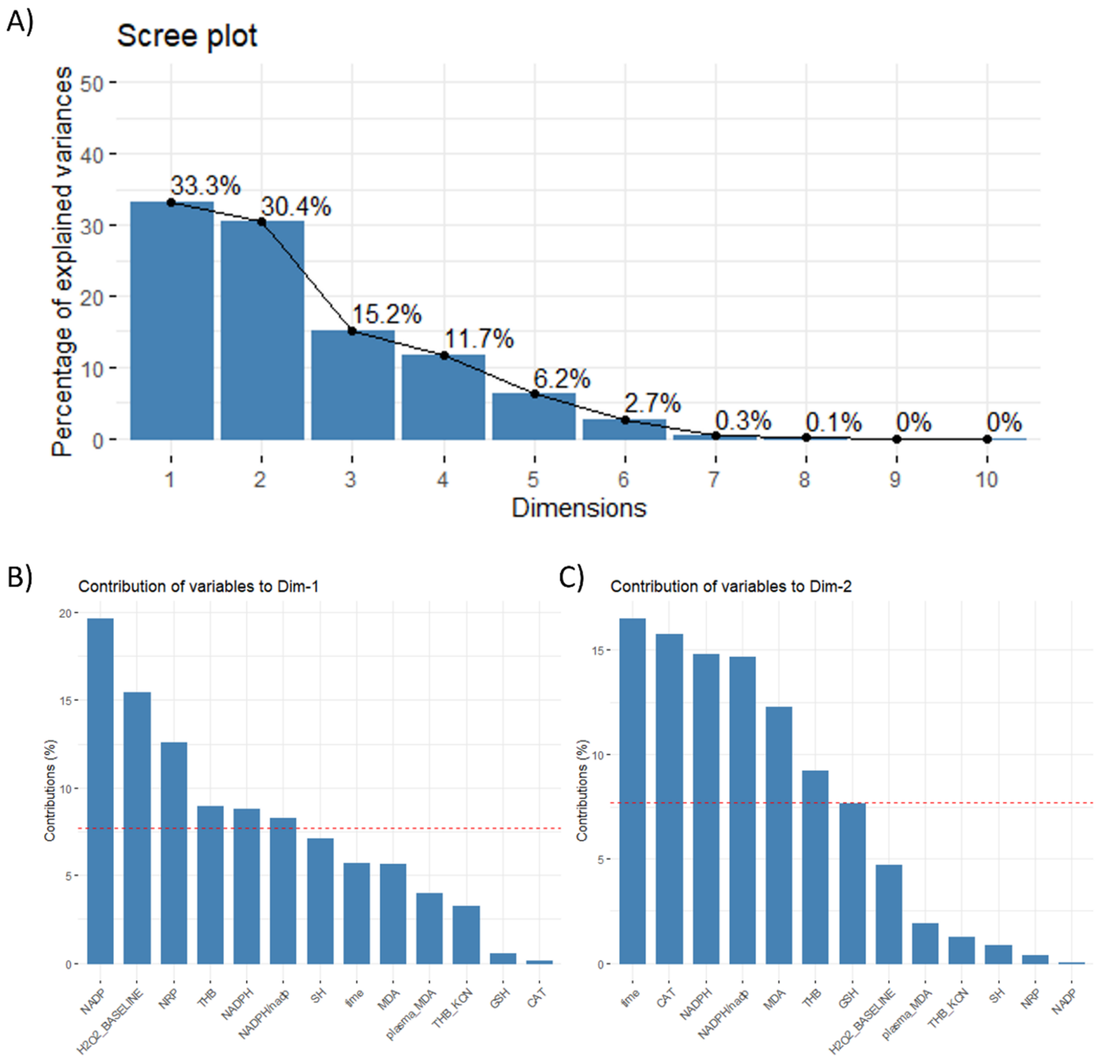

Figure S2. Duodenal redox-related parameters principal component analysis. (**A**) Scree plot. (**B**) Contribution of individual variables to the 1st principal component. (**C**) Contribution of individual variables to the 2nd principal component. NRP – nitrocellulose redox permanganoemtry; time – minutes since the exposure to treatment (continuous variable); THB_KCN – change in 1,2,3-trihydroxybenzene autooxidation rate in the presence of 2mM potassium cyanide (KCN); GSH – glutathione (the main representative of the low molecular weight thiols (LMWT)); MDA – malondialdehyde (the main representative of the thiobarbituric acid reactive substances (TBARS)); NADP – nicotinamide adenine dinucleotide phosphate; THB – change in 1,2,3-trihydroxybenzene autooxidation rate; CAT – catalase activity (hydrogen peroxide dissociation rate); H2O2_BASELINE – estimated baseline levels of tissue hydrogen peroxide; SH – protein reactive sulfhydryl groups; NADPH – reduced nicotinamide adenine dinucleotide phosphate; NADPH/nadp – the concentration of reduced nicotinamide adenine dinucleotide phosphate corrected for concentration of nicotinamide adenine dinucleotide phosphate; contrib – contribution of variables; Dim1 – 1st dimension; Dim2 – 2nd dimension.

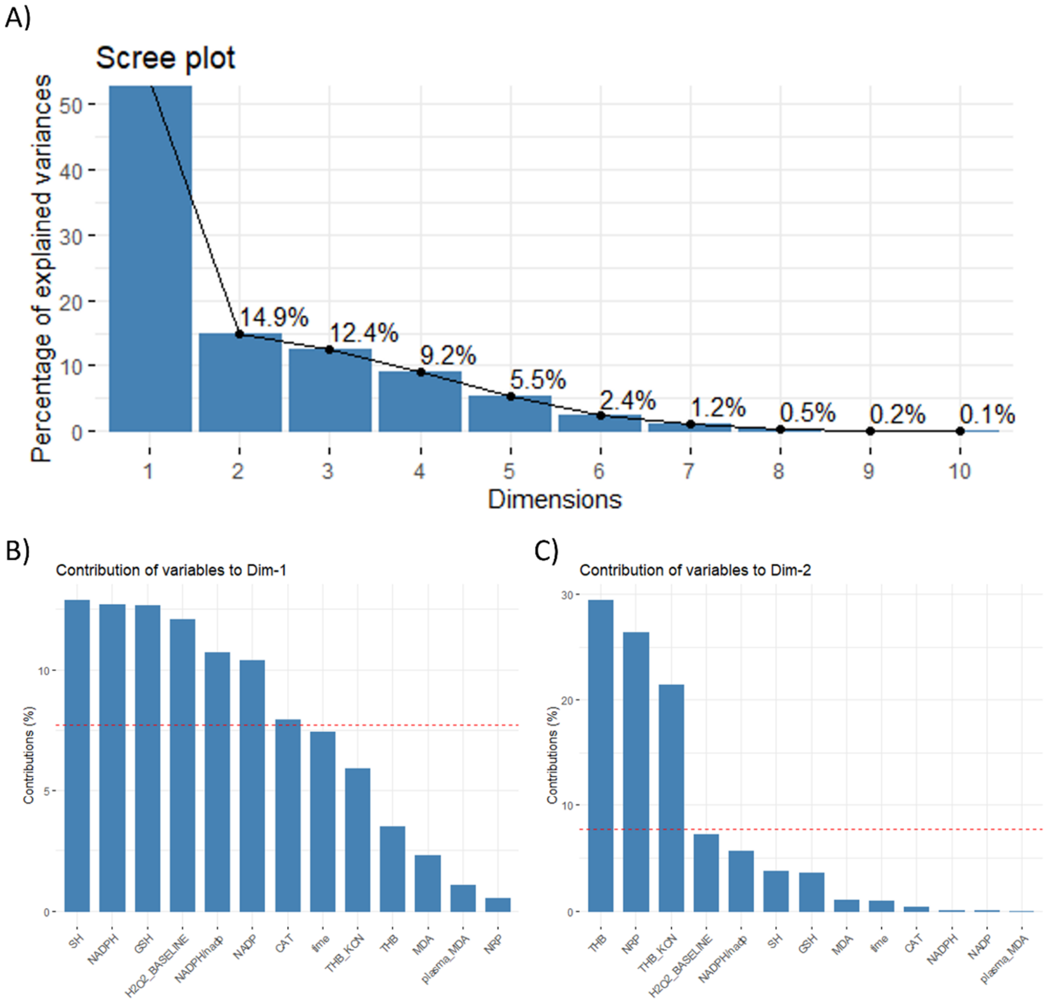

Figure S3. Ileal redox-related parameters principal component analysis. (**A**) Scree plot. (**B**) Contribution of individual variables to the 1st principal component. (**C**) Contribution of individual variables to the 2nd principal component. NRP – nitrocellulose redox permanganoemtry; time – minutes since the exposure to treatment (continuous variable); THB_KCN – change in 1,2,3-trihydroxybenzene autooxidation rate in the presence of 2mM potassium cyanide (KCN); GSH – glutathione (the main representative of the low molecular weight thiols (LMWT)); MDA – malondialdehyde (the main representative of the thiobarbituric acid reactive substances (TBARS)); NADP – nicotinamide adenine dinucleotide phosphate; THB – change in 1,2,3-trihydroxybenzene autooxidation rate; CAT – catalase activity (hydrogen peroxide dissociation rate); H2O2_BASELINE – estimated baseline levels of tissue hydrogen peroxide; SH – protein reactive sulfhydryl groups; NADPH – reduced nicotinamide adenine dinucleotide phosphate; NADPH/nadp – the concentration of reduced nicotinamide adenine dinucleotide phosphate corrected for concentration of nicotinamide adenine dinucleotide phosphate; contrib – contribution of variables; Dim1 – 1st dimension; Dim2 – 2nd dimension.
